# Crystal structure of 1-fluoro-1,3-di­hydro­benzo[*c*]thio­phene 2,2-dioxide

**DOI:** 10.1107/S2056989015016357

**Published:** 2015-09-12

**Authors:** Ying Zou, Zibin Qiu, Renming Tang, Kaixu Yuan, Ya Li

**Affiliations:** a Department of Chemistry and Chemical Engineering, Shanghai University of Engineering Science, 333 Longteng Road, Shanghai, People’s Republic of China

**Keywords:** crystal structure, sulfone, fluorine, di­hydro­benzo­thio­phene, C—H⋯O and C—H⋯F inter­actions

## Abstract

In the title compound, C_8_H_7_FO_2_S, the thio­phene ring has an envelope conformation, with the S atom bearing the two O atoms being the flap. In the crystal, mol­ecules are linked by C—H⋯O and C—H⋯F inter­actions, generating a three-dimensional network structure.

## Related literature   

For the use of of α-fluoro sulfones in organic synthesis, see: Fukuzumi *et al.* (2006 ▸); Li *et al.* (2006 ▸); Prakash *et al.* (2003 ▸); Zhao *et al.* (2013 ▸). For their synthesis, see: Jiang *et al.* (2014 ▸); Ni *et al.* (2008 ▸).
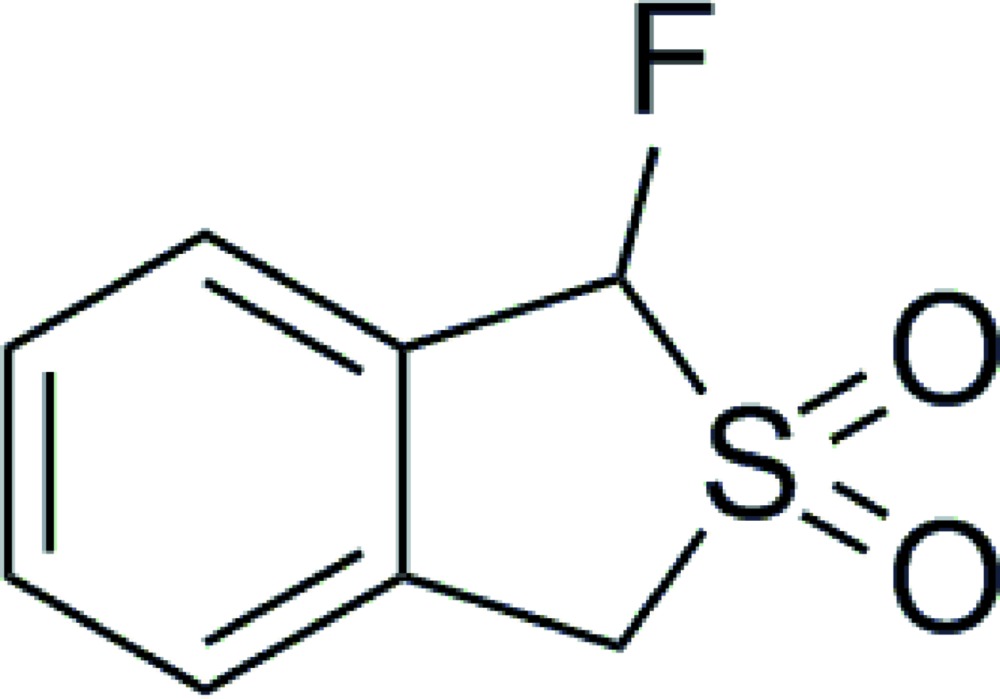


## Experimental   

### Crystal data  

C_8_H_7_FO_2_S
*M* *_r_* = 186.20
Monoclinic, 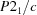

*a* = 5.7772 (5) Å
*b* = 8.3886 (6) Å
*c* = 16.8717 (12) Å
β = 99.742 (6)°
*V* = 805.86 (11) Å^3^
*Z* = 4
Cu *K*α radiation
μ = 3.38 mm^−1^
*T* = 296 K
0.05 × 0.03 × 0.02 mm

### Data collection  

Bruker APEXII CCD diffractometer
Absorption correction: multi-scan (*SADABS*; Bruker, 2009 ▸) *T*_min_ = 0.418, *T*_max_ = 0.753
6724 measured reflections
1475 independent reflections
1206 reflections with *I* > 2σ(*I*)
*R*_int_ = 0.080

### Refinement  

*R*[*F* ^2^ > 2σ(*F* ^2^)] = 0.068
*wR*(*F* ^2^) = 0.205
*S* = 1.13
1475 reflections
109 parameters
H-atom parameters constrained
Δρ_max_ = 0.58 e Å^−3^
Δρ_min_ = −0.98 e Å^−3^

### 

Data collection: *APEX2* (Bruker, 2009 ▸); cell refinement: *SAINT* (Bruker, 2009 ▸); data reduction: *SAINT*; program(s) used to solve structure: *SHELXS97* (Sheldrick, 2008 ▸); program(s) used to refine structure: *SHELXL2014* (Sheldrick, 2015 ▸); molecular graphics: *OLEX2* (Dolomanov *et al.*, 2009 ▸) and *Mercury* (Macrae, 2006 ▸); software used to prepare material for publication: *OLEX2*.

## Supplementary Material

Crystal structure: contains datablock(s) I. DOI: 10.1107/S2056989015016357/wm5205sup1.cif

Structure factors: contains datablock(s) I. DOI: 10.1107/S2056989015016357/wm5205Isup2.hkl

Supporting information file. DOI: 10.1107/S2056989015016357/wm5205Isup3.pdf

Supporting information file. DOI: 10.1107/S2056989015016357/wm5205Isup4.pdf

Supporting information file. DOI: 10.1107/S2056989015016357/wm5205Isup5.pdf

Supporting information file. DOI: 10.1107/S2056989015016357/wm5205Isup6.cml

**Fig. 1.**
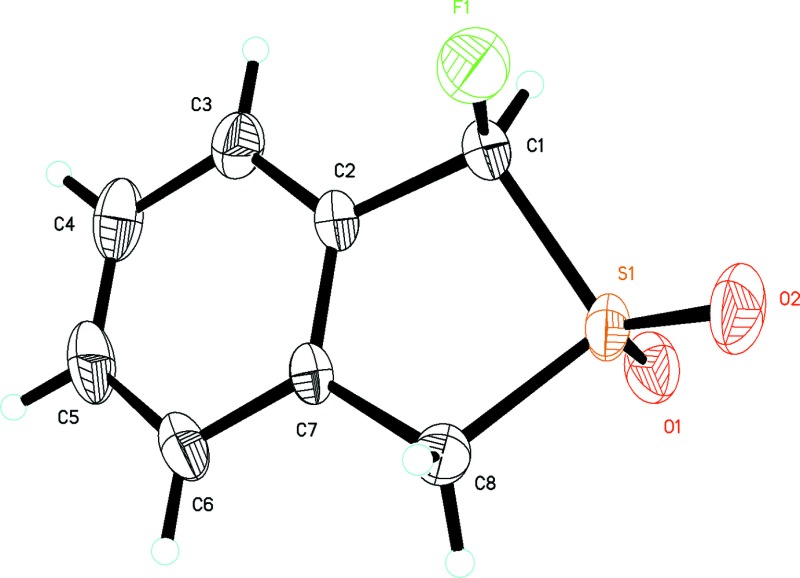
Molecular structure of the title compound. Displacement ellipsoids are drawn at the 50% probability level.

. DOI: 10.1107/S2056989015016357/wm5205fig1.tif
Mol­ecular structure of the title compound. Displacement ellipsoids are drawn at the 50% probability level.

**Fig. 2.**
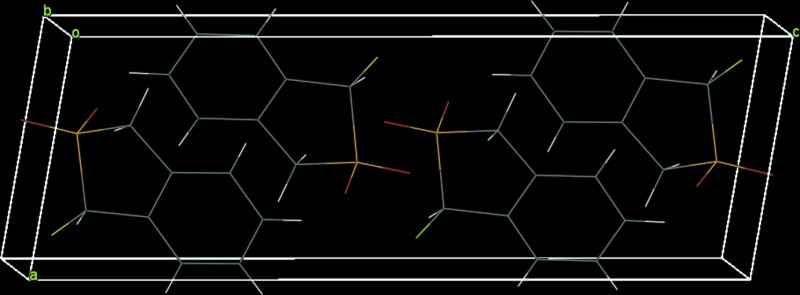
Packing of the molecules in the unit cell in a view approximately along [010].

. DOI: 10.1107/S2056989015016357/wm5205fig2.tif
Packing of the mol­ecules in the unit cell in a view approximately along [010].

CCDC reference: 1421889

Additional supporting information:  crystallographic information; 3D view; checkCIF report

## Figures and Tables

**Table 1:** Hydrogen-bond geometry (, )

*D*H*A*	*D*H	H*A*	*D* *A*	*D*H*A*
C3H3O1^i^	0.93	2.95	3.687(6)	138
C1H1O1^ii^	0.98	2.47	3.266(5)	139
C1H1O2^ii^	0.98	3.48	4.331(6)	147
C4H4O2^iii^	0.93	2.54	3.371(5)	148
C5H5F1^iv^	0.93	2.81	3.640(5)	150
C8H8*B*O2^v^	0.97	2.50	3.406(5)	156

Symmetry codes: (i) 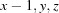; (ii) 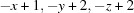; (iii) 
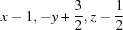
; (iv) 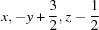; (v) 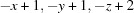.

## supplementary crystallographic information

### S1. Synthesis and crystallization

Lithium bis­(tri­methyl­silyl)amide (LiHMDS; 2.2 ml, 1.0 M in THF, 2.2 mmol, 2.2 equiv) and anhydrous ZnCl_2_ (341 mg, 2.5 mmol, 2.5 equiv) were dissolved in 12 ml THF. 10 minutes later, 1,3-di­hydro­benzo[*c*]thio­phene-2,2-dioxide (168 mg, 1.0 mmol, 1.0 equiv) was added into the mixture under N_2_ atmosphere. The reaction was stirred for one hour. Then *N*-fluoro­benzene­sulfonimide (NFSI; 2.0 equiv, 632 mg, 2.0 mmol) was added into the mixture in a flash. The reaction was allowed at room temperature for half an hour and quenched by addition of H_2_O. After extraction with ethyl acetate, the organic layer was dried over anhydrous Na_2_SO_4_, filtered and removed under vacuum. The crude product was purified by flash column chromatography on silica gel with ethyl acetate/hexane (1:3) to provide the title compound (97 mg, 52%). The obtained powder was recrystallized from di­chloro­methane/hexane (1:10) solution to give colourless crystals.

^1^H NMR (400 MHz, CDCl_3_) δ = 7.64-7.66 (d, *J* = 8.0 Hz, 1H), 7.56-7.59 (m, 1H), 7.48-7.52 (m, 1H), 7.36-7.38 (d, *J* = 8.0 Hz, 1H), 6.03 (d, *J* = 56.4 Hz, 1H), 4.40 (dd, *J* = 60.0, 16.0 Hz, 2H). ^19^F NMR (376 MHz, CDCl_3_) δ = -153.70 (d, *J* = 56.4 Hz). ^13^C NMR (101 MHz, CDCl_3_) δ = 128.6 (d, *J* = 4.0 Hz), 126.1, 125.5 (d, *J* = 4.0 Hz), 124.8, 122.5, 122.1, 94.2 (d, *J* = 86.4 Hz), 49.9. MS (EI) m/z: 168 [M + H - 19]^+^. HRMS (EI) m/z: calcd for C_8_H_8_O_2_S [M + H - 19]^+^ 168.0245, found 168.0251.

### S2. Refinement

All H atoms of the phenyl groups were placed at calculated positions and treated as riding on their parent atoms, with C—H = 0.93 Å and *U*_iso_(H) = 1.2*U*_eq_(C). The methyl­ene H atoms were found from a difference map. Their positions were refined with C—H = 0.97 Å and *U*_iso_(H) = 1.2*U*_eq_(C).

### Crystal data

**Table:**

C_8_H_7_FO_2_S	*F*(000) = 384
*M**_r_* = 186.20	*D*_x_ = 1.535 Mg m^−^^3^
Monoclinic, *P*2_1_/*c*	Cu *K*α radiation, λ = 1.54178 Å
*a* = 5.7772 (5) Å	Cell parameters from 1262 reflections
*b* = 8.3886 (6) Å	θ = 5.3–66.3°
*c* = 16.8717 (12) Å	µ = 3.38 mm^−^^1^
β = 99.742 (6)°	*T* = 296 K
*V* = 805.86 (11) Å^3^	Block, colourless
*Z* = 4	0.05 × 0.03 × 0.02 mm

### Data collection

**Table:**

Bruker APEXII CCD diffractometer	1206 reflections with *I* > 2σ(*I*)
Graphite monochromator	*R*_int_ = 0.080
φ and ω scans	θ_max_ = 69.6°, θ_min_ = 5.3°
Absorption correction: multi-scan (*SADABS*; Bruker, 2009)	*h* = −6→6
*T*_min_ = 0.418, *T*_max_ = 0.753	*k* = −10→9
6724 measured reflections	*l* = −19→17
1475 independent reflections	

### Refinement

**Table:**

Refinement on *F*^2^	Primary atom site location: structure-invariant direct methods
Least-squares matrix: full	Hydrogen site location: inferred from neighbouring sites
*R*[*F*^2^ > 2σ(*F*^2^)] = 0.068	H-atom parameters constrained
*wR*(*F*^2^) = 0.205	*w* = 1/[σ^2^(*F*_o_^2^) + (0.1242*P*)^2^ + 0.3031*P*] where *P* = (*F*_o_^2^ + 2*F*_c_^2^)/3
*S* = 1.13	(Δ/σ)_max_ < 0.001
1475 reflections	Δρ_max_ = 0.58 e Å^−^^3^
109 parameters	Δρ_min_ = −0.98 e Å^−^^3^
0 restraints	

### Special details

**Table:**

Geometry. All e.s.d.'s (except the e.s.d. in the dihedral angle between two l.s. planes) are estimated using the full covariance matrix. The cell e.s.d.'s are taken into account individually in the estimation of e.s.d.'s in distances, angles and torsion angles; correlations between e.s.d.'s in cell parameters are only used when they are defined by crystal symmetry. An approximate (isotropic) treatment of cell e.s.d.'s is used for estimating e.s.d.'s involving l.s. planes.

### Fractional atomic coordinates and isotropic or equivalent isotropic displacement parameters (Å^2^)

**Table:**

	*x*	*y*	*z*	*U*_iso_*/*U*_eq_	
S1	0.58177 (16)	0.76462 (12)	0.95887 (5)	0.0438 (4)	
F1	0.1554 (5)	0.6784 (4)	0.96561 (16)	0.0686 (8)	
O1	0.6976 (5)	0.9083 (4)	0.94354 (18)	0.0593 (9)	
O2	0.6325 (6)	0.6952 (5)	1.03759 (17)	0.0675 (10)	
C1	0.2672 (7)	0.7960 (5)	0.9290 (2)	0.0442 (9)	
H1	0.2194	0.9030	0.9431	0.053*	
C2	0.2387 (7)	0.7727 (4)	0.8397 (2)	0.0381 (8)	
C3	0.0537 (8)	0.8364 (5)	0.7852 (3)	0.0521 (10)	
H3	−0.0632	0.8964	0.8027	0.063*	
C4	0.0492 (10)	0.8075 (6)	0.7037 (3)	0.0633 (13)	
H4	−0.0734	0.8477	0.6661	0.076*	
C5	0.2237 (10)	0.7203 (6)	0.6781 (3)	0.0638 (13)	
H5	0.2178	0.7029	0.6233	0.077*	
C6	0.4093 (8)	0.6576 (5)	0.7325 (2)	0.0512 (10)	
H6	0.5276	0.5994	0.7147	0.061*	
C7	0.4136 (6)	0.6840 (4)	0.8142 (2)	0.0373 (8)	
C8	0.6028 (7)	0.6252 (5)	0.8808 (2)	0.0442 (9)	
H8A	0.7564	0.6286	0.8649	0.053*	
H8B	0.5711	0.5175	0.8969	0.053*	

### Atomic displacement parameters (Å^2^)

**Table:**

	*U*^11^	*U*^22^	*U*^33^	*U*^12^	*U*^13^	*U*^23^
S1	0.0448 (6)	0.0535 (7)	0.0294 (6)	−0.0075 (4)	−0.0046 (4)	−0.0040 (3)
F1	0.0590 (15)	0.098 (2)	0.0493 (15)	−0.0118 (15)	0.0102 (11)	0.0065 (13)
O1	0.0623 (18)	0.0629 (19)	0.0519 (16)	−0.0229 (15)	0.0075 (13)	−0.0147 (14)
O2	0.068 (2)	0.095 (2)	0.0328 (16)	−0.0078 (18)	−0.0109 (14)	0.0109 (15)
C1	0.047 (2)	0.054 (2)	0.0302 (19)	−0.0005 (17)	0.0026 (14)	−0.0082 (15)
C2	0.045 (2)	0.0384 (19)	0.0283 (18)	−0.0040 (15)	−0.0003 (14)	−0.0037 (13)
C3	0.053 (2)	0.051 (2)	0.047 (2)	0.0017 (18)	−0.0075 (17)	0.0000 (17)
C4	0.078 (3)	0.063 (3)	0.038 (2)	−0.013 (2)	−0.019 (2)	0.0103 (19)
C5	0.092 (4)	0.069 (3)	0.027 (2)	−0.021 (3)	−0.001 (2)	−0.0043 (18)
C6	0.068 (3)	0.052 (2)	0.0358 (19)	−0.0143 (19)	0.0140 (17)	−0.0135 (16)
C7	0.0453 (19)	0.0335 (18)	0.0313 (17)	−0.0072 (14)	0.0011 (14)	−0.0070 (13)
C8	0.0427 (19)	0.043 (2)	0.045 (2)	0.0018 (16)	0.0020 (15)	−0.0037 (16)

### Geometric parameters (Å, º)

**Table:**

S1—O1	1.423 (3)	C3—C4	1.392 (6)
S1—O2	1.434 (3)	C4—H4	0.9300
S1—C1	1.821 (4)	C4—C5	1.373 (8)
S1—C8	1.781 (4)	C5—H5	0.9300
F1—C1	1.381 (5)	C5—C6	1.391 (7)
C1—H1	0.9800	C6—H6	0.9300
C1—C2	1.502 (5)	C6—C7	1.392 (5)
C2—C3	1.393 (5)	C7—C8	1.512 (5)
C2—C7	1.381 (5)	C8—H8A	0.9700
C3—H3	0.9300	C8—H8B	0.9700
			
O1—S1—O2	118.9 (2)	C3—C4—H4	119.6
O1—S1—C1	107.8 (2)	C5—C4—C3	120.7 (4)
O1—S1—C8	109.19 (19)	C5—C4—H4	119.6
O2—S1—C1	110.6 (2)	C4—C5—H5	119.4
O2—S1—C8	112.9 (2)	C4—C5—C6	121.3 (4)
C8—S1—C1	94.61 (18)	C6—C5—H5	119.4
S1—C1—H1	112.0	C5—C6—H6	120.8
F1—C1—S1	107.1 (3)	C5—C6—C7	118.4 (4)
F1—C1—H1	112.0	C7—C6—H6	120.8
F1—C1—C2	112.1 (3)	C2—C7—C6	120.2 (4)
C2—C1—S1	101.1 (3)	C2—C7—C8	114.8 (3)
C2—C1—H1	112.0	C6—C7—C8	125.0 (4)
C3—C2—C1	123.6 (4)	S1—C8—H8A	111.4
C7—C2—C1	114.9 (3)	S1—C8—H8B	111.4
C7—C2—C3	121.4 (4)	C7—C8—S1	101.8 (2)
C2—C3—H3	121.0	C7—C8—H8A	111.4
C4—C3—C2	118.0 (4)	C7—C8—H8B	111.4
C4—C3—H3	121.0	H8A—C8—H8B	109.3
			
S1—C1—C2—C3	−156.5 (3)	C1—C2—C7—C8	0.2 (5)
S1—C1—C2—C7	22.5 (4)	C2—C3—C4—C5	−0.7 (7)
F1—C1—C2—C3	89.7 (5)	C2—C7—C8—S1	−23.5 (4)
F1—C1—C2—C7	−91.2 (4)	C3—C2—C7—C6	0.7 (6)
O1—S1—C1—F1	−162.2 (2)	C3—C2—C7—C8	179.3 (4)
O1—S1—C1—C2	80.4 (3)	C3—C4—C5—C6	0.3 (8)
O1—S1—C8—C7	−79.0 (3)	C4—C5—C6—C7	0.6 (7)
O2—S1—C1—F1	−30.6 (3)	C5—C6—C7—C2	−1.0 (6)
O2—S1—C1—C2	−148.1 (3)	C5—C6—C7—C8	−179.6 (4)
O2—S1—C8—C7	146.3 (3)	C6—C7—C8—S1	155.1 (3)
C1—S1—C8—C7	31.6 (3)	C7—C2—C3—C4	0.2 (6)
C1—C2—C3—C4	179.3 (4)	C8—S1—C1—F1	86.0 (3)
C1—C2—C7—C6	−178.4 (3)	C8—S1—C1—C2	−31.5 (3)

### Hydrogen-bond geometry (Å, º)

**Table:**

*D*—H···*A*	*D*—H	H···*A*	*D*···*A*	*D*—H···*A*
C3—H3···O1^i^	0.93	2.95	3.687 (6)	138
C1—H1···O1^ii^	0.98	2.47	3.266 (5)	139
C1—H1···O2^ii^	0.98	3.48	4.331 (6)	147
C4—H4···O2^iii^	0.93	2.54	3.371 (5)	148
C5—H5···F1^iv^	0.93	2.81	3.640 (5)	150
C8—H8*B*···O2^v^	0.97	2.50	3.406 (5)	156

Symmetry codes: (i) *x*−1, *y*, *z*; (ii) −*x*+1, −*y*+2, −*z*+2; (iii) *x*−1, −*y*+3/2, *z*−1/2; (iv) *x*, −*y*+3/2, *z*−1/2; (v) −*x*+1, −*y*+1, −*z*+2.
