# Supplementary figures and images for: Crystal structure of 1-fluoro-1,3-di­hydro­benzo[c]thio­phene 2,2-dioxide
Source: Acta Crystallogr E Crystallogr Commun. 2015 Sep 12;71(Pt 10):o749. doi: 10.1107/S2056989015016357 (PMC4647347; doi:10.1107/S2056989015016357)

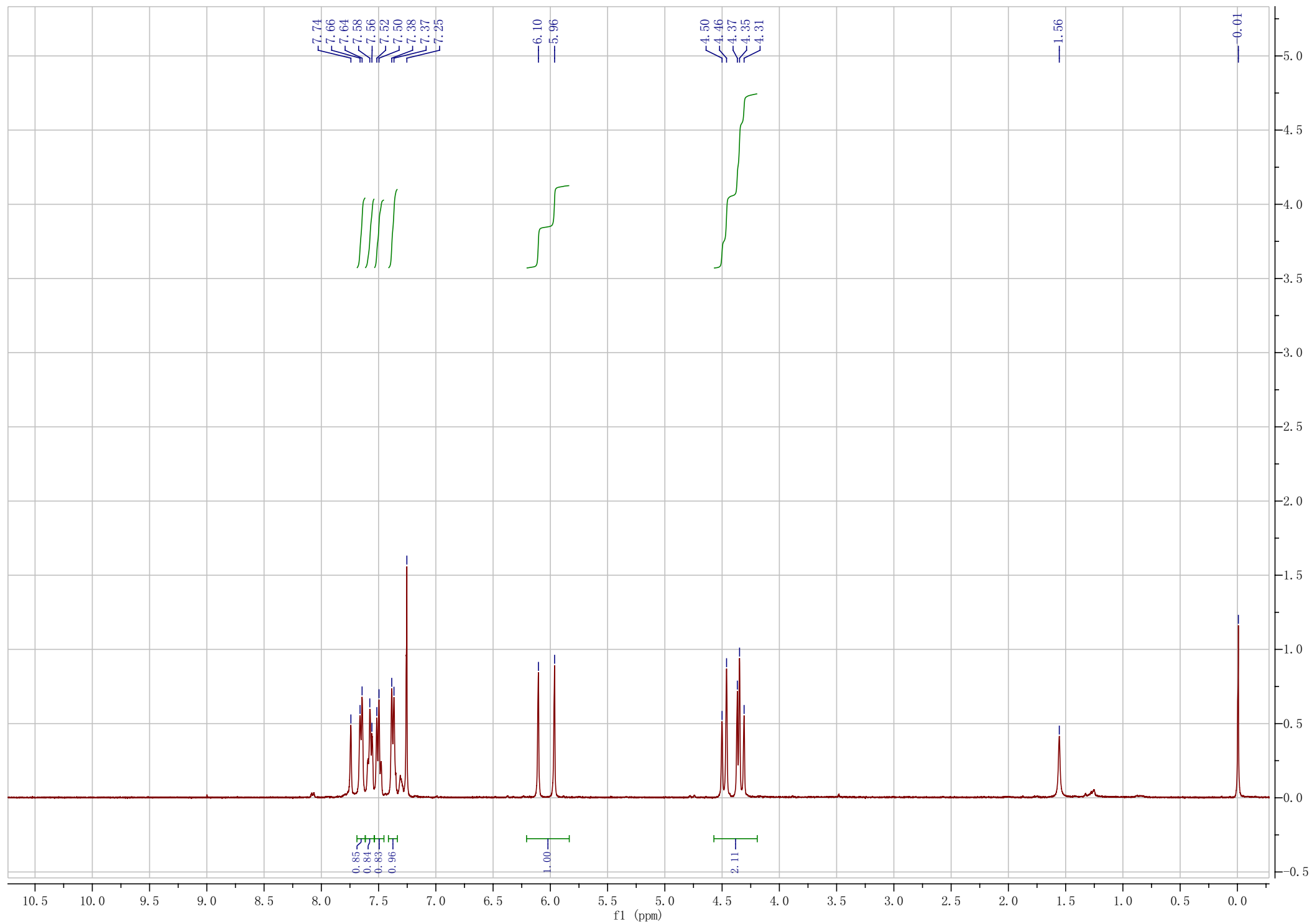

Supplement: Supplementary file 3 [file e-71-0o749-Isup3.pdf]

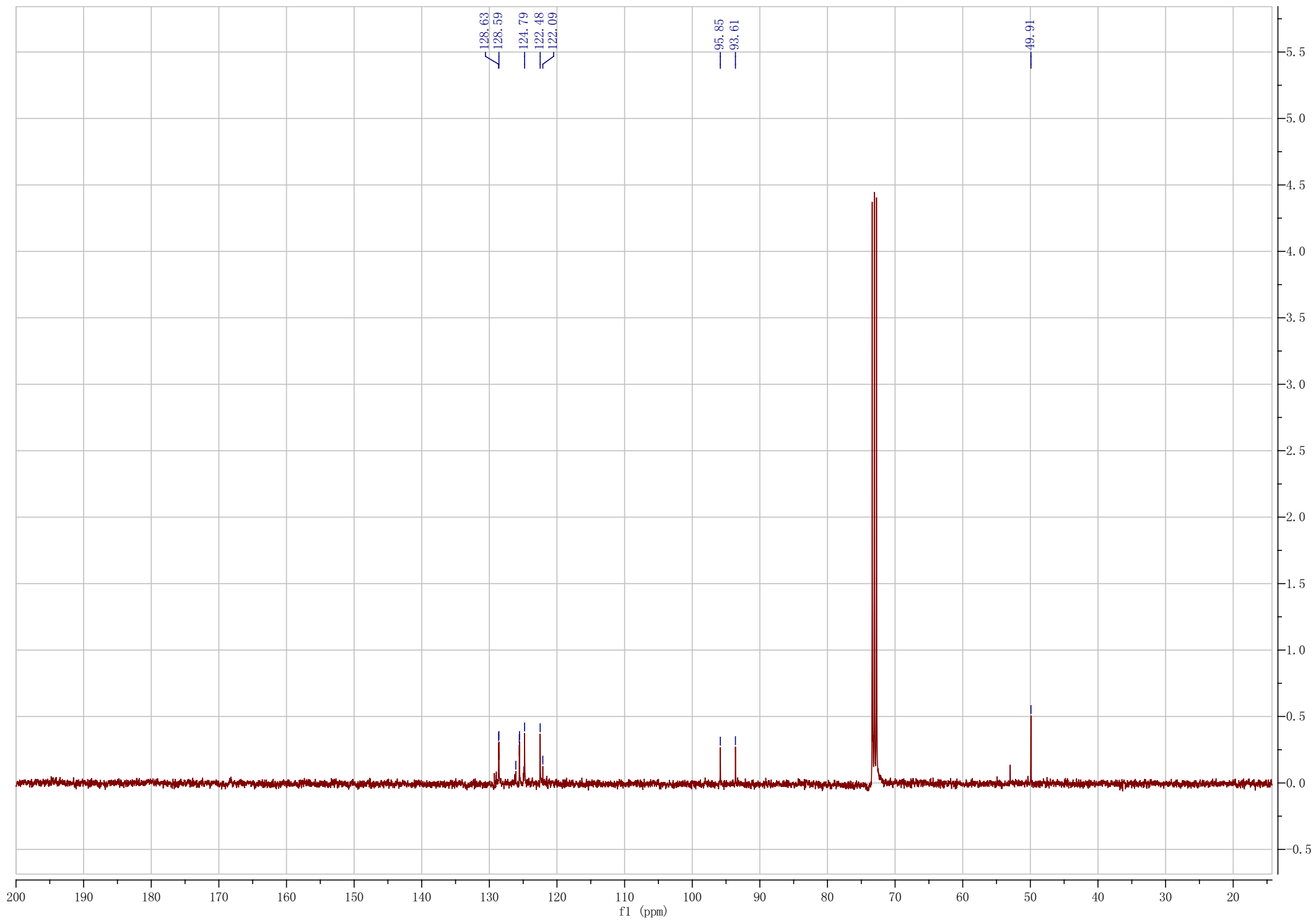

Supplement: Supplementary file 4 [file e-71-0o749-Isup4.pdf]

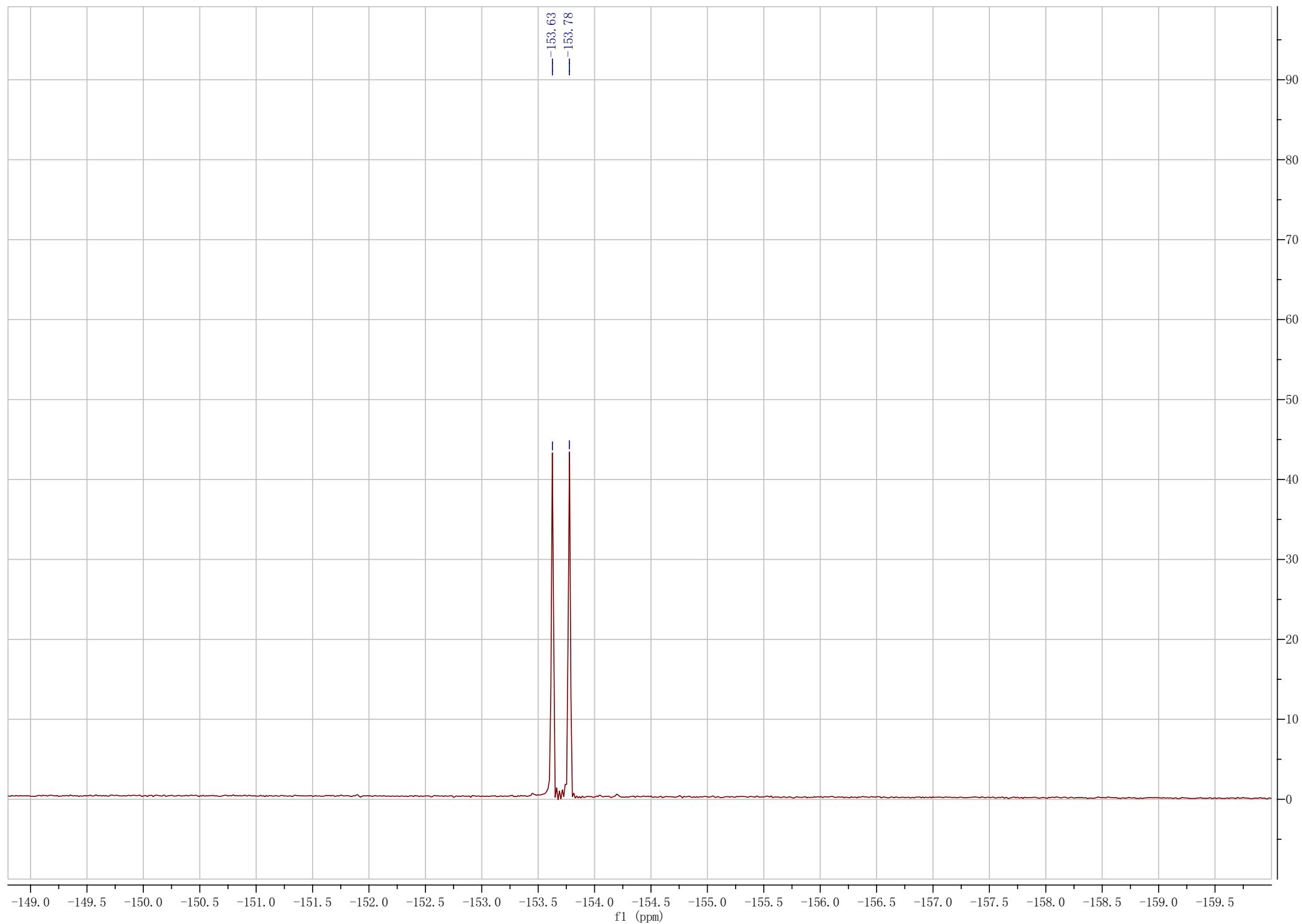

Supplement: Supplementary file 5 [file e-71-0o749-Isup5.pdf]

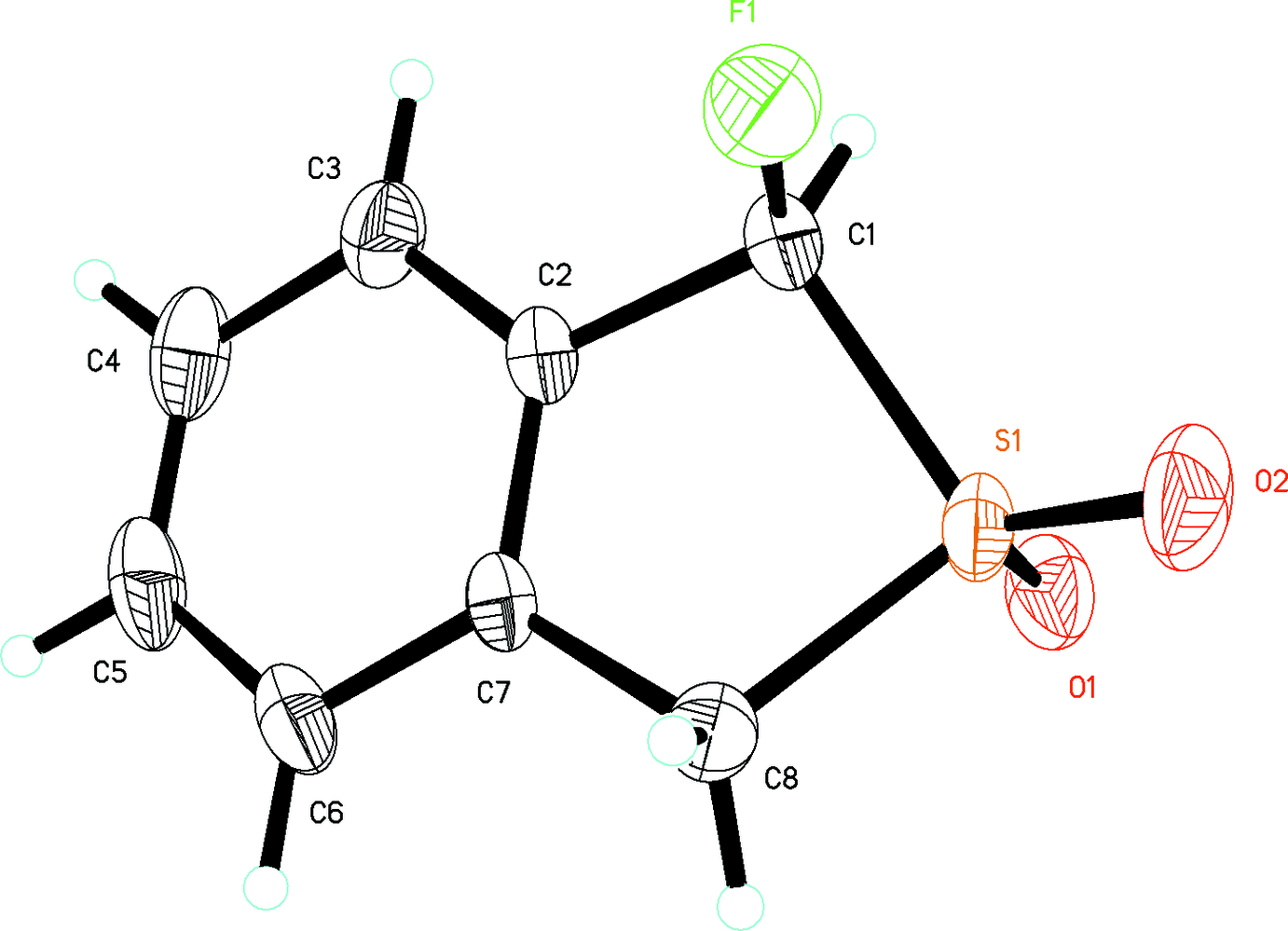

Supplement: Supplementary file 7 [file e-71-0o749-fig1.tif]

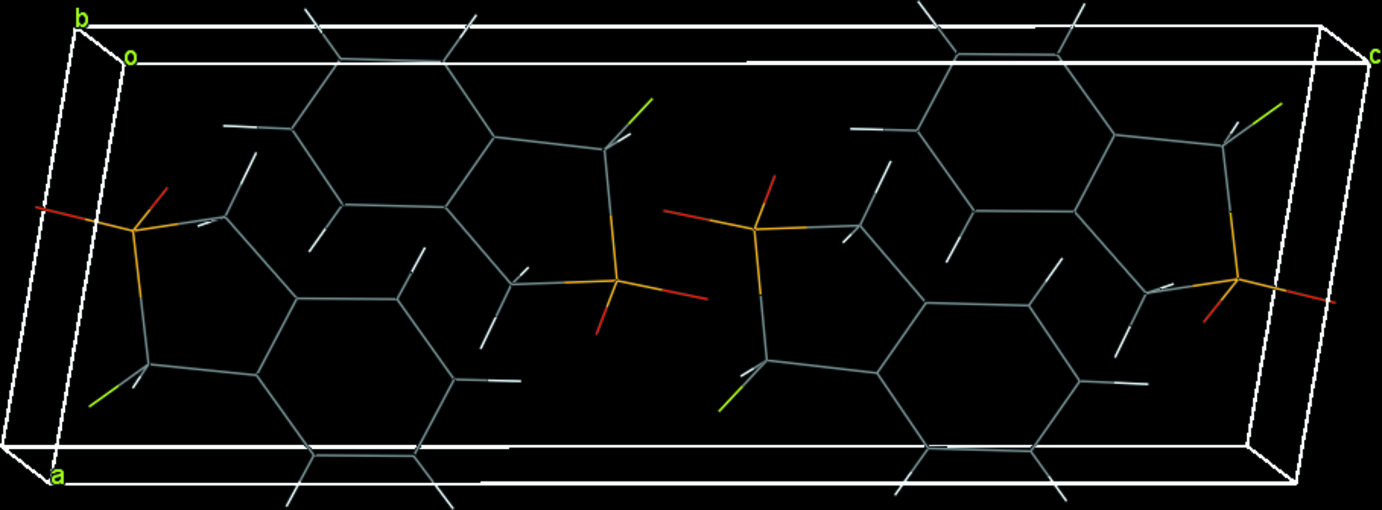

Supplement: Supplementary file 8 [file e-71-0o749-fig2.tif]
